# Different Clinical Presentations in Eating Disorder Patients with Non-Suicidal Self-Injury Based on the Co-Occurrence of Borderline Personality Disorder

**DOI:** 10.5334/pb.420

**Published:** 2018-09-27

**Authors:** Laurence Claes, Brianna Turner, Eva Dierckx, Koen Luyckx, Margaux Verschueren, Katrien Schoevaerts

**Affiliations:** 1 Faculty of Psychology and Educational Sciences, KU Leuven, Leuven, BE; 2 Faculty of Medicine and Health Sciences, University Antwerp, Antwerp, BE; 3 Department of Psychology, University of Victoria, Victoria, BC, CA; 4 Department of Psychology, Harvard University, Cambridge, MA, US; 5 Psychiatric Hospital Alexianen Tienen, Tienen, BE; 6 Department of Psychology and Educational Sciences, Vrije Universiteit Brussel, Brussels, BE; 7 UNIBS, University of the Free State, Bloemfontein, ZA

**Keywords:** eating disorder, borderline personality, self-harm, psychopathology

## Abstract

Non-suicidal self-injury (NSSI) and borderline personality disorder (BPD) features are common in patients with eating disorders (ED), yet little is known regarding the clinical presentation of ED patients who present with NSSI with and without BPD. The current study compared self-injurious, female ED inpatients with (n = 98; NSSI+BPD) and without BPD (n = 45; NSSI-only) on different self-reported clinical features. Results suggest that ED patients with NSSI+BPD differ from those with NSSI-only with regard to frequency of suicidal ideation, alcohol, drug or medication abuse, internalizing/externalizing psychopathology, interpersonal problems, and coping strategies, with the NSSI+BPD group demonstrating more impairment in each of these domains. Despite these differences in clinical presentation, however, groups did not differ in NSSI features. In sum, while self-injurious ED patients may present with similar NSSI behavior regardless of BPD diagnosis, those with NSS+BPD represent a group with much higher clinical complexity and greater treatment needs.

## Introduction

Non-suicidal self-injury (NSSI) refers to the direct injury of one’s own body tissue without suicidal intent, such as cutting, scratching, and hitting oneself (Claes & Vandereycken, 2007). Up till the publication of the fifth edition of the *Diagnostic and Statistical Manual of Mental Disorders* (DSM-5; APA, 2013), NSSI was solely listed as a possible symptom of the borderline personality disorder (BPD). However, given the accumulating research evidence that shows that NSSI can also occur in patients without BPD (e.g., Glenn & Klonsky, 2013; Nock et al., 2006; Selby, Kranzler, Fehling, & Panza, 2015; Turner et al., 2015), NSSI was proposed as a separate diagnosis in the DSM-5, as a Condition for Further Study (APA, 2013). To distinguish the clinical presentation of NSSI in populations with and without a diagnosis of BPD, research assessing both NSSI and BPD symptoms is urgently needed in order to clarify the presentation and treatment needs of these populations.

Only recently, Turner et al. (2015) compared self-injurious adults with NSSI-only and NSSI+BPD on NSSI features and co-occurring psychiatric symptoms in a community sample. Community adults with NSSI+BPD engaged in more frequent, recent, and severe NSSI than those with NSSI-only. Adults with NSSI+BPD also reported significantly more anxiety disorders, but rates of mood, substance or psychotic disorders did not differ from the NSSI-only group. Finally, adults with NSSI+BPD reported more depressive symptoms, suicidal ideation, and emotion regulation difficulties than the self-injurious adults without BPD. Few studies aside from Turner’s (2015) study have compared NSSI in patients with NSSI+BPD and NSSI-only. Some studies have shown that inpatients with NSSI+BPD use a larger number of NSSI methods and have made more suicide attempts compared to inpatients with NSSI-only (Anestis et al., 2015; Turner et al., 2013; Turner et al., 2015). Similarly, inpatients with NSSI+BPD engaged more often in self-scratching and overdosing compared to inpatients with NSSI-only (Sansone et al., 2005). As far as we know, no studies have directly compared clinical symptoms between inpatients with NSSI-only and NSSI+BPD. Therefore, one of the aims of the present study was to compare NSSI and clinical features of inpatients with NSSI-only and those with NSSI+BPD. Given that NSSI and BPD are both highly prevalent in inpatients with eating disorders (ED), this patient sample was selected to address the aforementioned question and to partially fill this gap in the existing literature.

The prevalence of NSSI is estimated between 25.4% and 55.2% in patients with ED (Claes & Muehlenkamp, 2014; Svirko & Hawton, 2007), and more specifically between 14% and 42% in patients with anorexia nervosa (AN), restrictive type; and between 26% and 55% in patients with bulimia nervosa (BN; Svirko & Hawton, 2007). Furthermore, many authors have reported that ED patients with NSSI report higher levels of clinical symptomatology, personality disorder features, trauma, and inadequate coping strategies, compared to ED patients without NSSI (Claes & Muehlenkamp, 2014; Claes, Vandereycken, & Vertommen, 2001, 2003).

Similarly, many studies have shown comorbidity between ED and personality disorders (Ro et al., 2005; Vrabel et al., 2010). Most (75%) ED inpatients have one or more personality disorders (Rosenvinge, Martinussen, & Ostensen, 2000). The most common personality disorders in ED were avoidant and dependent (Cluster C), as well as borderline (Cluster B) personality disorders (Bornstein, 2001). In more recent meta-analyses, Cluster C personality disorders were most commonly diagnosed among AN restrictive subtype (Cassin & von Ranson, 2005; Farstad, McGeown, & Von Ranson, 2016). Individuals with BN and AN bingeing subtype are more often diagnosed with Cluster B and C personality disorders (Cassin & von Ranson, 2005; Farstad, McGeown, & Von Ranson, 2016). According to several authors (e.g., Ro et al., 2005; Zanarini et al., 2010), personality disorders may diminish treatment response and elevate the risk of poor treatment outcome in ED patients. Finally, the risk for NSSI doubled and the risk for suicide attempts increased fivefold in ED patients with comorbid BPD (Nutzinger & Andreas, 2008).

The present study aimed to characterize NSSI features, clinical symptoms, personality disorders symptoms, and coping strategies in adult self-injurious ED patients with NSSI-only and NSSI+BPD. Based on prior studies, both in population and clinical samples, we expected more severe and more versatile NSSI behaviors in the NSSI+BPD group compared to the NSSI-only group (Anestis et al., 2015; Turner et al., 2013; Turner et al., 2015). With respect to clinical symptomatology, we expected more severe ED psychopathology, more anxiety and depression, and more severe suicidal thoughts and behaviors in the NSSI+BPD group (Sansone et al., 2005; Turner et al., 2015). With respect to personality disorders, we expected more severe symptoms of Cluster A, B, and C personality psychopathology in the NSSI+BPD group, except for the Obsessive-Compulsive personality disorder (OCPD), given the low association between BPD and OCPD (Zanarini et al., 1998). Furthermore, with respect to trauma and coping, we expected more traumatic experiences and less adequate coping strategies in the NSSI+BPD than in the NSSI-only group.

## Method

### Participants and procedure

Data were collected from the clinical records of inpatients treated in a specialized Eating Disorder unit for females in Belgium between June 2011 and March 2016. The current sample included all consecutively admitted female patients who were at least 18 years old and who had completed all of the questionnaires assessing NSSI, ED, and personality disorder symptomatology. All patients filled out the questionnaires during the first week of their admission, as part of the routine assessment procedure at the treatment unit. In total, we received data from 277 ED patients, of which 13 patients were excluded due to missing data on either the Eating Disorder Evaluation Scale (EDES; Vandereycken, 1993) or the Self-Injury Questionnaire – Treatment Related (SIQ-TR; Claes & Vandereycken, 2007), and 2 patients were removed due to an age less than 18 years. A further 119 patients did not engage in NSSI, leaving a sample of 143 (55%) ED patients who displayed at least one act of NSSI during their lifetimes.

Of these 143 self-injurious ED patients, 98 (68.5%) fulfilled the criteria for a categorical BPD diagnosis as assessed by a well-validated self-report questionnaire (see Assessment DSM-IV Personality Disorders, below), whereas 45 inpatients (31.5%) did not. The mean number of met BPD criteria for the former ED group (NSSI+BPD) was 7.04 (*SD* = 1.42), whereas it was 2.62 (*SD* = 1.13) for the latter ED group (NSSI-only) [*F*(1,141) = 336.34, *p* < .001]. The mean age of the total sample was 23.15 years (*SD* = 5.41), with no significant difference in age between the NSSI+BPD (*M* = 22.63, *SD* = 4.99) and NSSI-only (*M* = 24.29, *SD* = 6.13) groups [*F*(1,141) = 2.931, *ns*].

Of the 143 ED patients, 29 (20.3%) were diagnosed as anorexia nervosa (AN), restrictive type, 42 (29.4%) as AN, binge-eating/purging type, 47 (32.9%) as bulimia nervosa, and 25 (17.5%) as ED, not otherwise specified. There was no significant association between ED subtype and BPD diagnosis in the self-injurious ED sample [χ^2^_(3)_ = 2.554, *ns*], so we did not control for ED subtype while analyzing the data.

During all phases of the study, we adhered to the provisions of the Declaration of Helsinki. Patients gave written informed consent at admission to anonymously use their data for scientific purposes by signing an informed consent form and this procedure was approved by the medical-ethical committee of the clinic in which the research was performed.

### Instruments

The Self-Injury Questionnaire-Treatment Related (SIQ-TR; Claes & Vandereycken, 2007) investigates the presence/absence of five different methods of NSSI (i.e., scratching, cutting, burning, hitting, and biting oneself). For each method of NSSI, we asked how long ago the patient had engaged in this form of NSSI (a week ago, a month ago, several months ago, more than a year ago, and never). In the present study, we focused on the presence/absence of NSSI over the lifetime (i.e., NSSI was considered to be present if at least one of the five NSSI behaviors was displayed during lifetime; α = .73) and on the presence/absence of recent NSSI (i.e., NSSI was considered to be recently present if at least one of the five NSSI behaviors was displayed during the last week/month). NSSI versatility refers to the total number of different NSSI methods (ranging from 1 to 5) that were displayed by the patients recently (a week/month ago) and over the lifetime. NSSI versatility can be considered as an index of NSSI severity (Sleuwaegen et al., 2017).

The Eating Disorder Inventory-2 (EDI-2; Garner, 1991) is a reliable and valid inventory assessing several behavioral and psychological traits common in ED. The EDI-2 has 11 subscales: Drive for Thinness (α = .92), Bulimia (α = .93), Body Dissatisfaction (α = .92), Ineffectiveness (α = .87), Perfectionism (α = .76), Interpersonal Distrust (α = .83), Lack of Interoceptive Awareness (α = .69), Maturity Fears (α = .88), Asceticism (α = .68), Impulse Dysregulation (α = .74), and Social Insecurity (α = .74). To calculate the eleven subscale scores, the scores on each item belonging to the particular subscale are summed. Higher scores indicate more severe ED psychopathology.

To assess psychopathology, we used the Symptom Checklist (SCL-90; Dutch version: Arrindell & Ettema, 1986). The SCL-90 is a widely used measure of affective and interpersonal psychiatric symptoms. It consists of 90 items (symptoms) to be rated on a five-point scale ranging from ‘not at all applicable’ to ‘strongly applicable’. Along with a global scale of psychopathology (α = .97), it measures symptoms of general anxiety (α = .88), phobic anxiety (α = .80), depression (α = .88), somatization (α = .84), obsessions/ compulsions (α = .85), paranoid ideation and interpersonal sensitivity (α = .90), hostility (α = .77), sleeplessness (α = .74), and psychoticism (α = .74). The SCL-90 has shown ‘good’ to ‘very good’ concurrent, convergent, discriminant, and construct validity (Arrindell & Ettema, 1986). Given that the SCL-90 has no items related to externalizing symptomatology, we added one item of the Eating Disorder Examination Scale (EDES; Vandereycken, 1993) to investigate the presence/absence of alcohol, drugs, or medication abuse.

Categorical and dimensional personality disorder scores were measured using the Assessment of DSM-IV Personality Disorders (ADP-IV; Schotte et al., 1998). The 94 items of the ADP-IV assess the 80 criteria of the 10 DSM-IV personality disorders. Each item was assessed on a seven-point scale (1 = totally disagree, 7 = totally agree) (Trait score). For each trait that was rated 5 or higher (rather agree), participants also rated on a three-point scale how much distress that symptom causes the patient or others (1 = not at all, 3 = most certainly) (Distress score). Dimensional trait scores were computed by adding the trait scores within the 10 personality disorders. The categorical diagnostic evaluation followed the DSM-IV personality disorder definition by combining the trait and distress scores in scoring algorithms (e.g., each criterion scored as present if the Trait score was > 4 and the Distress score was > 1). The ADP-IV dimensional scales demonstrated good internal consistency in this study, with alpha coefficients between .63 (schizoid personality disorder) and .87 (avoidant personality disorder). The Cronbach’s alpha coefficient for the borderline personality disorder was .77.

Coping strategies were assessed with the Utrecht Coping List (UCL; Schreurs et al., 1993). Each of the 47 items of the UCL are rated on a four-point scale. The seven subscales assess the following coping strategies: Active Problem Solving (α = .81), Palliative Reactions (α = .59), Passive/Depressive Reactions (α = .71), Avoidance (α = .57), Social Support Seeking (α = .89), Expression of Emotions (α = .59), and Self-Soothing Thoughts (α = .72). Previous research supports the UCL’s reliability and construct validity (Schreurs et al., 1993).

The Traumatic Experiences Questionnaire (TEQ; Nijenhuis, van der Hart, & Kruger, 2002) evaluates the following adverse and traumatic experiences: emotional neglect and abuse, physical abuse, sexual abuse (by family members and others), serious family problems (such as alcohol abuse, poverty), loss or death of a family member, bodily harm, and war experiences. Each of 29 possible events is rated as present or absent. The TEQ had good internal consistency in the present study (α = .78). Considerable evidence supports the reliability and validity of the TEQ in clinical populations (Nijenhuis, Van der Hart, & Kruger, 2002).

### Analyses

To compare ED patients with NSSI+BPD versus those with NSSI-only, we used MANOVAs with affective and interpersonal symptomatology, personality disorder dimensional scores, coping strategies, and number and type of traumatic events as dependent variables and BPD diagnosis (yes/no) in self-injurious ED patients as the independent variable. Associations between BPD diagnosis and other dichotomous variables (e.g., medication use, alcohol or drugs abuse) were calculated by means of the Chi-Square Test Statistic. All analyses were performed by means of IBM SPSS Statistics 24.

A post-hoc power analysis performed using G*Power 3.1 (Faul, Erdfelder, Buchner, & Lang, 2009) indicated excellent power (1 – β = .99) for multivariate tests assuming a large effect, but more modest power to detect medium effects (1 – β = .61 to .75). For chi-square comparisons, the current sample provided excellent power to detect medium (1 – β = .95) as well as large effects (1 – β = .99).

## Results

### NSSI and suicidal ideation in ED patients with NSSI+BPD and NSSI-only

Of the total self-injurious ED sample, 70 (49%) patients engaged in recent NSSI (last week to last month), whereas 73 (51%) did not. The NSSI+BPD group did not significantly differ from the NSSI-only group with respect to NSSI recency (χ^2^_(1)_ = .00, *ns*; NSSI-only [48.9%] vs. NSSI+BPD [49%]). In the same vein, we did not find significant group differences with respect to lifetime NSSI versatility [NSSI-only (*M* = 2.13, *SD* = 1.16) vs. NSSI+BPD (*M* = 2.46, *SD* = 1.22), *F*(1,141) = 3.27, *ns*] or recent NSSI versatility [NSSI-only (*M* = 1.59, *SD* = 1.01) vs. NSSI+BPD (*M* = 1.77, *SD* = .81), *F*(1,68) = .40, *ns*]. Similar results were found for rates of engagement in each of the five NSSI methods separately (i.e., scratching, biting, cutting, burning and head banging). However, on a single item assessing frequency of suicidal ideation [SCL-90 item 59, scored from 1 (never) to 5 (always)], ED patients with NSSI+BPD (*M* = 2.78, *SD* = 1.45) scored significantly higher than patients with NSSI-only (*M* = 2.22, *SD* = 1.13), *F*(1,141) = 5.10, *p* < .05.

### Severity of eating disorder, affective and interpersonal symptoms in ED patients with NSSI+BPD and NSSI-only

Table 1 displays the means and standard deviations of the EDI-2 scales in ED patient with NSSI-only and NSSI+BPD. Overall, we found significant differences between the groups [Wilks’ Lambda = .666, *F*(11, 125) = 5.69, *p* < .001]. ED patients with NSSI+BPD scored significantly higher on Bulimia, Impulse Dysregulation, Ineffectiveness, Lack of Interoceptive Awareness, Interpersonal Distrust, and Social Insecurity scales compared to ED patients with NSSI-only.

**Table 1:** Means (standard deviations) of the EDI-2 scales for self-injurious ED patients without and with borderline personality disorder (BPD).

EDI-2	NSSI-only (n = 42)		NSSI+BPD (n = 95)		*F*(1,135)		Partial η^2^

	*M*	*(SD)*	*M*	*(SD)*			

Drive for Thinness	32.69	(8.42)	35.14	(7.07)	3.09		.02
Bulimia	20.14	(8.27)	23.84	(9.87)	4.50	*	.03
Body Dissatisfaction	43.24	(9.98)	45.84	(8.39)	2.49		.02
Ineffectiveness	42.07	(8.45)	47.01	(6.88)	12.99	**	.09
Perfectionism	23.43	(5.40)	23.96	(5.75)	.26		.00
Interpersonal Distrust	24.86	(5.66)	27.86	(5.69)	8.16	**	.06
Lack of Interoceptive Awareness	35.71	(6.85)	41.60	(5.62)	27.85	***	.17
Maturity Fears	28.93	(7.77)	29.53	(7.99)	.17		.00
Asceticism	30.33	(5.98)	32.29	(5.52)	3.49		.03
Impulse Dysregulation	27.88	(4.58)	36.12	(6.77)	51.63	***	.28
Social Insecurity	29.64	(6.11)	32.72	(4.90)	9.79	**	.07

*p < .05, **p < .01, ***p < .001.

Concerning affective and interpersonal symptomatology (SCL-90), ED patients with NSSI+BPD scored significantly higher on all clinical symptom scales compared to patients with NSSI-only [Wilks’ Lambda = .788, *F*(9, 127) = 3.79, *p* < .001] (see Table 2). The strongest differences were found on the General Psychopathology, Anxiety, Interpersonal Sensitivity, and Psychoticism scales.

**Table 2:** Means (standard deviations) of the SCL-90 scales for self-injurious ED patients without and with borderline personality disorder (BPD).

SCL-90	NSSI-only (n = 42)		NSSI+BPD (n = 95)		*F*(1,135)		Partial η^2^

	*M*	*(SD)*	*M*	*(SD)*			

Agoraphobia	12.45	(3.81)	16.51	(6.08)	15.85	***	.11
Anxiety	23.90	(7.58)	31.23	(8.29)	23.96	***	.15
Depression	46.64	(11.55)	54.74	(11.87)	13.76	***	.09
Somatisation	27.45	(7.88)	32.16	(9.25)	8.21	**	.06
Insufficiency (OCD)	22.62	(7.98)	27.31	(7.55)	10.84	***	.07
Interpersonal sensitivity	41.74	(11.82)	52.53	(13.14)	20.84	***	.13
Hostility	9.48	(2.92)	12.53	(4.70)	15.05	***	.10
Sleeping problems	8.48	(3.71)	9.8	(3.32)	4.30	*	.03
Psychoticism	20.45	(5.19)	25.57	(6.44)	20.58	***	.13
Global Psychopathology	213.21	(48.73)	262.36	(56.31)	24.01	***	.15

*p < .05, **p < .01, ***p < .001.

We also investigated whether the presence/absence of alcohol, drugs, or medication abuse (EDES item) was significantly related to the presence/absence of BPD in self-injurious ED patients (given that the SCL-90 has no items related to addiction). We found that ED patients with NSSI+BPD had a significant higher probability to engage in alcohol, drugs, or medication abuse (38.8%) compared to ED patients with NSSI-only (17.8%) [χ^2^_(1)_ = 6.231, *p* < .05].

### Personality disorders scores of ED patients with NSSI+BPD and NSSI-only

With respect to personality disorder scores (ADP-IV) significant differences appeared between the NSSI-only and the NSSI+BPD group [Wilks’ Lambda = .436, *F*(12, 124) = 13.371, *p* < .001] (see Table 3). The ED patients with NSSI+BPD scored significantly higher on all Cluster A, Cluster B, and Cluster C personality disorder dimensional scales compared to ED patients with NSSI-only, except for the Cluster C-Obsessive-Compulsive personality disorder scale. The effect sizes were small for the Cluster A-Schizoid and Cluster C-Active-Avoidant Personality Disorders. The strongest effect sizes were found for the Cluster B BPD, Histrionic Personality Disorder and the Passive-Aggressive Personality Disorder. Similar results were found if we used the categorical personality disorders scores instead of the dimensional scores.

**Table 3:** Means (standard deviations) of the dimensional ADP-IV scales for self-injurious ED patients without and with borderline personality disorder (BPD).

ADP-IV	NSSI-only (n = 43)		NSSI+BPD (n = 94)		*F*(1,135)		Partial η^2^

	*M*	*(SD)*	*M*	*(SD)*			

Cluster A	62.81	(16.12)	82.80	(18.09)	38.46	***	.22
Paranoid	19.12	(5.99)	28.01	(8.08)	41.58	***	.23
Schizoid	20.77	(6.25)	23.23	(6.82)	4.06	*	.03
Schizotypal	22.93	(7.32)	31.55	(8.22)	34.72	***	.21
Cluster B	86.23	(14.16)	124.29	(22.87)	101.07	***	.43
BPD	34.42	(6.46)	50.65	(7.23)	158.73	***	.54
Histrionic	19.98	(4.28)	29.56	(7.26)	64.54	***	.32
Antisocial	13.86	(4.46)	20.18	(7.34)	27.21	***	.17
Narcissistic	17.98	(6.01)	23.89	(8.32)	17.51	***	.12
Cluster C	87.81	(19.79)	102.10	(19.43)	15.75	***	.10
Avoidant	28.95	(9.14)	33.53	(8.27)	8.46	**	.06
Dependent	27.11	(6.71)	34.03	(7.86)	24.83	***	.16
Obsessive-Compulsive	31.74	(7.23)	34.53	(8.24)	3.64		.03
Depressive	27.40	(6.56)	34.21	(6.59)	31.64	***	.19
Passive-Aggressive	16.46	(3.84)	24.11	(6.96)	45.49	***	.25

*p < .05, **p < .01, ***p < .001.

### Coping styles in ED patients with NSSI+BPD and NSSI-only

Descriptive information for the coping scores (UCL) for ED patients with NSSI+BPD and NSSI-only is displayed in Table 4. Overall, we found significant differences between the groups with respect to coping styles [Wilks’ Lambda = .825, *F*(7, 135) = 4.10, *p* < .001]. The results clearly show that ED patients with NSSI+BPD scored significantly lower on Active Problem Solving behaviors and significantly higher on Depressive (Passive) Reaction Patterns compared to ED patients with NSSI-only. The other subscales did not reveal significant differences between both ED groups.

**Table 4:** Means (standard deviations) of the UCL scales for self-injurious ED patients without and with borderline personality disorder (BPD).

UCL	NSSI-only (n = 45)		NSSI+BPD (n = 98)		*F*(1,141)		Partial η^2^

	*M*	*(SD)*	*M*	*(SD)*			

Active Problem Solving	16.22	(4.18)	14.13	(3.90)	8.46	**	.06
Palliative Reaction Pattern	20.09	(3.38)	20.38	(3.54)	0.21		.00
Avoidance	17.87	(2.40)	18.61	(3.61)	1.59		.01
Social Support	11.76	(3.75)	11.59	(3.95)	.06		.00
(Depressive) Reaction Pattern	15.87	(3.38)	19.00	(3.60)	24.30	***	.15
Expression of Emotions	6.00	(1.87)	6.35	(2.00)	.97		.01
Self-soothing thoughts	12.20	(3.14)	11.37	(3.09)	2.21		.02

*p < .05, **p < .01, ***p < .001.

### Traumatic experiences in ED patients with NSSI+BPD and NSSI-only

Finally, we did not find significant differences between the total number of traumatic events between ED patients with NSSI+BPD (*M* = 5.03, *SD* = 3.91) and ED patients with NSSI-only (*M* = 4.24, *SD* = 3.70), [*F*(1, 141) = 1.29, *ns*]. In the same vein, both groups did not differ significantly from each other if we compared them for each type of traumatic experiences separately.

## Discussion

Consistent with past studies (Svirko & Hawton, 2007), the present study documents a high prevalence of NSSI (around 55%) in ED inpatients, underscoring the importance of examining variability in clinical presentations among patients who present with both of these behaviors. Of the self-injurious ED patients, 68.5% met BPD criteria, with a mean number of BPD symptoms of 7.04. The self-injurious group without BPD diagnosis was clearly differentiated form the BPD group, with a mean of BPD criteria of 2.62. This indicates the presence of two distinct groups of self-injurious ED patients: those who present with NSSI in the absence of significant BPD symptoms (NSSI-only), and those who present with NSSI and BPD symptoms (NSSI+BPD). Our analyses examined additional distinctions between these groups, in order to clarify the presentation and treatment needs of these populations.

Contrary to our expectations, ED patients with NSSI+BPD did not differ from those with NSSI-only with respect to the recency, severity, versatility, or methods of their NSSI. This indicates that BPD symptomatology does not predict a more severe or persistent type of NSSI behavior in ED patients. While previous comparisons of self-injurious community adults with and without BPD found differences in NSSI frequency, recency, and severity (Turner et al., 2015), neither study found differences in NSSI methods. This suggests that in a more clinically severe population (i.e., ED inpatients), NSSI behavior may present as a more uniform phenomenon. Why we did not find significant differences in the presentation of NSSI between ED patients with NSSI-only and NSSI+BPD is not clear, and certainly deserves further study. With respect to suicidal ideation, however, the current results show that ED patients with NSSI+BPD endorsed more frequent thoughts of death compared to ED patients with NSSI-only. This is in line with findings of previous studies in patient (e.g., Sansone et al., 2002, 2005), community (Turner et al., 2015), and population samples (Klonsky & Olino, 2008) showing more severe suicidal behaviors in subpopulations with BPD. This higher level of suicidal ideation could possibly be linked to the higher levels of emotional symptomatology (anxiety and depression related symptoms) in the NSSI+BPD compared to the NSSI-only group. Several studies in clinical samples (e.g., Claes et al., 2010), have shown that patients who engage in both NSSI and suicidal ideation report higher levels of clinical symptomatology and less adequate coping styles compared to patients who engage in NSSI-only or suicidal ideation-only.

In addition to higher scores on internalizing symptomatology (e.g., depression, anxiety) in ED patients with NSSI+BPD, we also found elevations in externalizing/impulsive symptomatology (e.g., bulimia, alcohol/drugs/medication abuse) and interpersonal symptomatology (hostility, interpersonal distrust, social discomfort) as compared to ED patients with NSSI-only. These findings are completely in line with prior studies (e.g., Galione & Zimmerman, 2010) which show that BPD comorbidity in other axis I disorders increase the probability of showing more internalizing and externalizing symptomatology. In the same vein, ED patients with NSSI+BPD also scored significantly higher on all dimensional personality disorder scales compared to those with NSSI-only, except for the Cluster C obsessive-compulsive personality disorder. The latter personality disorder, which is often more prevalent in restrictive ED patients (e.g., Farstad, McGeown, & Von Ranson, 2016), is characterized by anxiety and also by controlled behavior, which is not in line with the emotional dysregulated and impulsive nature of the often more bulimic ED patients with BPD features (Turner, Claes et al., 2014). Therefore, future studies in larger samples, should take ED subtype into account while comparing personality features in ED patients with NSSI-only and NSSI+BPD. Furthermore, and contrary to our expectations, we did not find a significant difference in the total number of experienced traumata between self-injurious ED patients with and without BPD. Most studies so far (e.g., Utzinger et al., 2016), showed higher levels of traumatic events in ED patients with BPD compared to those without BPD. However, by selecting an ED sample, of whom all patients engaged in NSSI (often related to trauma) made the variability in traumatic events in ED patients with and without BPD features probably less clear. However, this hypothesis needs further investigation. Finally, NSSI+BPD patients also reported significantly higher scores on a passive depressive coping style and significantly lower scores on an active problem solving coping style compared to ED patients with NSSI-only. NSSI+BPD patients seem to be less able to actively cope with the problems they meet; and rather seem to react in an emotional, passive-depressive way, which concurs with their higher levels of depressive symptomatology and depressive personality features.

Based on these findings, the treatment of ED patients with NSSI+BPD should take into account the complexity of the multiple presenting symptoms. In a recent meta-analysis, Farstad et al. (2015) suggest that ED patients characterized by emotional instability and impulsivity (like NSSI+BPD) may be better treated using integrative cognitive-affective therapy (ICAT) or dialectical behavior therapy (DBT), instead of cognitive behavioral therapy for ED (CBT-E), given that ICAT and DBT teach skills related to distress tolerance and emotion regulation (p. 100). These emotion regulation difficulties and ineffective coping responses are likely to be at the core of the clinical complexity in such patients. Support for such a treatment matching approach is given by Accurso et al. (2016), who showed that BN patients with elevated levels of sensation seeking and/or emotional instability reported greater reductions in bulimic symptoms after receiving ICAT versus CBT-E (Accurso et al., 2016). Also psychodynamic-oriented interventions (e.g., mentalization based therapy) have shown significant improvements in impulsivity-related symptoms, as well as mood and interpersonal functioning in (ED) patients with BPD features (e.g., Fonagy & Bateman, 2006; Robinson et al., 2014). On the other hand, in patients who present with ED and NSSI but who do not meet criteria for BPD, a more focused and structured treatment such as CBT-E may help to reduce the core symptoms by emphasizing self-monitoring, exposure and behavioral activation.

Despite the strengths of our study, the study is not without limitations which merit further discussion. First, the study was performed in a sample of female ED patients who were admitted to a specialized treatment unit for ED. Therefore, this sample is limited to women with severe and acute psychopathology meriting inpatient treatment. Future studies should include male as well as female inpatients, and should compare clinical presentations of outpatients with NSSI-only versus NSSI+BPD. Secondly, the heterogeneity of our self-injurious ED sample may also have influenced our results. Future research in larger samples should certainly control for ED subtype while investigating differences between ED patients with NSSI-only and NSSI+BPD. Thirdly, there was no information available about the illness duration and prior hospitalization of the self-injurious ED patients, which could have influenced our findings. Therefore, these variables certainly need to be included in future studies. Fourthly, all variables were assessed by means of self-report questionnaires, which can overestimate the prevalence of some symptomatology (e.g., personality disorder features) and can elevate the association between variables due to shared method variance. Therefore, future studies should include different assessment tools (e.g., a standardized interview to assess personality disorders) as well as a multi-informant assessors. Fifthly, the multiple statistical comparisons in this study increased the risk for Type I error. We used multivariate statistics to reduce Type I error rates. Replication of these preliminary findings in other pathological groups (e.g., a group of depressed patients) is needed.

Despite these limitations, this study contributes to our knowledge regarding the clinical characteristics of two distinct groups of self-injurious ED patients: those with and without a diagnosis of BPD. Identifying these individuals has important implications for clinical practice. Moreover, the current findings highlight the importance of including information regarding BPD features in future research that seeks to clarify the association between ED symptoms and NSSI, as these diagnoses commonly overlap. To the extent that study samples include a large portion of patients with BPD, research findings may be biased by the relatively more severe clinical presentation of these patients compared to the patients without a BPD diagnosis. Together, then, we hope the current study can help to inform and refine practice and research in this important population.

In sum, we can conclude that the ED patients with NSSI+BPD show significantly higher levels of Axis I internalizing and externalizing symptoms and more suicidal ideation, more personality disorder features (besides BPD) and less active (adequate) and more passive (inadequate) coping styles compared to ED patients with NSSI-only, which was in line with our expectations. However, contrary to our expectations, we did not find significant differences in NSSI presentation and traumatic experiences between ED patients with and without BPD, which will be topics for future research.
